# Direct comparison of two different mesalamine formulations for the induction of remission in patients with ulcerative colitis: A double-blind, randomized study

**DOI:** 10.1002/ibd.21193

**Published:** 2010-01-04

**Authors:** Hiroaki Ito, Mitsuo Iida, Takayuki Matsumoto, Yasuo Suzuki, Hidetaka Sasaki, Toyomitsu Yoshida, Yuichi Takano, Toshifumi Hibi

**Affiliations:** * Digestive Disease Center of Excellence, Kitano Hospital, The Tazuke Kofukai Medical Research InstituteOsaka, Japan; † Department of Medicine and Clinical Science, Graduate School of Medical Science, Kyushu UniversityFukuoka, Japan; ‡ Division of Lower Gastroenterology, Department of Internal Medicine, Hyogo College of MedicineHyogo, Japan; § Department of Internal Medicine, Toho University Sakura Medical CenterChiba, Japan; ∥ Clinical Research, ZERIA Pharmaceutical Co., Ltd.Tokyo, Japan; ¶ Division of Gastroenterology and Hepatology, Department of Internal Medicine, Keio University School of MedicineTokyo, Japan

**Keywords:** mesalamine, ulcerative colitis, randomized controlled trial, colonoscopy

## Abstract

**Background:**

Mesalamine is the first-line drug for the treatment of ulcerative colitis (UC). We directly compared the efficacy and safety of two mesalamine formulations for the induction of remission in patients with UC.

**Methods:**

In a multicenter, double-blind, randomized study, 229 patients with mild-to-moderate active UC were assigned to 4 groups: 66 and 65 received a pH-dependent release formulation of 2.4 g/day (pH-2.4 g) or 3.6 g/day (pH-3.6 g), respectively; 65 received a time-dependent release formulation of 2.25 g/day (Time-2.25 g), and 33 received placebo (Placebo). The drugs were administered three times daily for eight weeks. The primary endpoint was a decrease in the UC disease activity index (UC-DAI).

**Results:**

In the full analysis set (*n* = 225) the decrease in UC-DAI in each group was 1.5 in pH-2.4 g, 2.9 in pH-3.6 g, 1.3 in Time-2.25 g and 0.3 in Placebo, respectively. These results demonstrate the superiority of pH-3.6 g over Time-2.25 g (*P* = 0.003) and the noninferiority of pH-2.4 g to Time-2.25 g. Among the patients with proctitis-type UC, a significant decrease in UC-DAI was observed in pH-2.4 g and pH-3.6 g as compared to Placebo, but not in Time-2.25 g. No differences were observed in the safety profiles.

**Conclusions:**

Higher dose of the pH-dependent release formulation was more effective for induction of remission in patients with mild-to-moderate active UC. Additionally, the pH-dependent release formulation was preferable to the time-dependent release formulation for patients with proctitis-type UC (UMIN Clinical Trials Registry, no. C000000288). (Inflamm Bowel Dis 2010)

Oral mesalamine formulations are widely used for the treatment of mild-to-moderate active ulcerative colitis (UC) because they have shown excellent efficacy and safety, especially with long-term use.^1^–^3^

Mesalamine is absorbed in the upper gastrointestinal tract;^3^–^5^ whereas, it exerts antiinflammatory activity directly on the inflamed mucosa in colon and rectum. Thus, many types of release-controlled oral formulations of mesalamine have been developed to enhance its effect.

The most widely-used formulation of mesalamine, the pH-dependent release formulation coated with Eudragit-S (Asacol), has been designed so that the coating film dissolves at a pH of 7 or higher.^3^,^6^,^7^ The release of mesalamine starts in the terminal ileum due to its coating. The time-dependent release formulation of mesalamine coated with ethyl cellulose (Pentasa), on the other hand, gradually releasing mesalamine in the stomach.^3^,^7^ Both formulations are designed to increase drug delivery to the inflamed areas of the colon and rectum as compared with the unmodified formulation. Since there have been no double-blind, randomized studies comparing the therapeutic efficacy of these formulations, physicians select these drugs with little guidance as to their proper use. Therefore, we conducted a double-blind, randomized trial in patients with active UC comparing the two formulations in terms of their efficacy and safety (UMIN Clinical Trials Registry, no. C000000288).

## MATERIALS AND METHODS

### Patient Selection

We conducted the study on patients with mild-to-moderate active UC on the basis of two inclusion criteria: 1) outpatients who were 16–64 years of age at the time of informed consent, and 2) patients who had mild-to-moderate active UC defined by UC-DAI of 3–8 and a bloody stool score of 1 or greater. The UC-DAI was originally developed by Sutherland et al.^8^

The patients were excluded according to the following criteria: 1) severe UC, chronic continuous type UC or acute fulminating type UC; 2) oral mesalamine more than 2.25 g daily, oral salazosulfapyridine more than 4.5 g daily, mesalamine enemas, salazosulfapyridine suppositories, corticosteroids (oral preparations, enemas, suppositories, injections and/or remedies for hemorrhoidal diseases) and/or cytapheresis within 14 days before the start of the investigational drug; 3) immunosuppressants within 90 days before the start of the investigational drug; 4) any other investigational drug within six months before informed consent; 5) a history of hypersensitivity to mesalamine or salicylate drugs, severe cardiac disease, severe pulmonary disease and/or severe hematological diseases; 6) severe hepatopathy, severe nephropathy and/or malignant tumors; and 7) pregnant or lactating.

### Ethical Considerations

This study was conducted according to the principles of the Declaration of Helsinki after obtaining approvals from the Institutional Review Board at each of the participating medical centers. Written informed consent was obtained from all participants.

### Study Drugs

The pH-dependent release mesalamine formulation used in this study is a tablet coated with Eudragit-S (Asacol 400 mg tablet, Tillotts Pharma AG, Ziefen, Switzerland, supplied by ZERIA Pharmaceutical, Tokyo, Japan). The time-dependent release mesalamine formulation used in this study is a tablet coated with ethyl cellulose (Pentasa 250 mg tablet, Nisshin Kyorin Pharmaceutical, Japan). This study was conducted using a double-dummy method.

### Study Design

This double-blind, randomized, controlled study was conducted at 53 centers in Japan. Treatment assignments were balanced according to the inflamed areas (proctitis-type or others) and the severity of the disease (range of UC-DAI at initial assessment: 3–5 or 6–8) with the use of a biased-coin minimization algorithm. Balance within each medical center was also taken into consideration. A person independent from the study was in charge of the random allocation. Seven patients were assigned as a block as follows: 2 to a group given the pH-dependent release formulation at 2.4 g/day (pH-2.4 g), 2 to a group given the pH-dependent release formulation at 3.6 g/day (pH-3.6 g), 2 to a group given the time-dependent release formulation at 2.25 g/day (Time-2.25 g), and 1 to a group given placebo (Placebo). The randomization code was sealed and stored until the blind was removed.

At the time of informed consent, investigators evaluated the background characteristics of patients. After an observation period of 3–14 days from the time of informed consent, investigators assessed patients for their eligibility for enrolment according to criteria previously described. At the time of the eligibility assessment the UC-DAI was calculated using a previously reported method.^9^,^10^ The UC-DAI is the sum of the mucosal appearance score (based on the colonoscopy findings by reference to atlases of mucosal appearance), stool frequency score, bloody stool score, and physician's global assessment score (stage 0, 1, 2, or 3). Each score was based on the patients' diary for the last three days. The area of the inflammation was also determined by colonoscopy. Patients who were judged as eligible were enrolled and assigned to investigational drugs by a central registration center, and then administration was started. The investigational drugs were administered three times daily for eight weeks.

During the study, each patient recorded the condition of their bloody stools, stool frequency and drug compliance in their diary and visited the medical center every two weeks. Each component of UC-DAI, except the mucosal appearance score, was assessed at each visit. Colonoscopy was performed at eight weeks or at withdrawal from the study, and UC-DAI was calculated at that time. To evaluate safety, clinical laboratory data and vital signs were checked at the time of informed consent and four weeks and eight weeks after enrolment (or upon withdrawal). The presence or absence of adverse events (AEs) and adverse drug reactions (ADRs) were recorded by investigators at each visit.

### Statistical Analysis

In the statistical analysis, the primary endpoint was the decrease in UC-DAI at the final assessment. The principal aim of this study was to demonstrate two hypotheses with closed procedure; the first was the superiority of pH-3.6 g over Time-2.25 g, and the second was the noninferiority of pH-2.4 g to Time-2.25 g.

In the primary endpoint, a closed procedure was adopted. Individual hypotheses were verified by the following methods: 1) verification of the superiority of pH-3.6 g over Time-2.25 g: the superiority was demonstrated if the lower limit of the 95% confidence interval (CI) was more than “0.0” in the difference of the decrease in UC-DAI between the groups (pH-3.6 g minus Time-2.25 g); and 2) verification of the noninferiority of pH-2.4 g to Time-2.25 g: the noninferiority and superiority would be demonstrated if the lower limits of the 95% CI were more than “−1.0” and “0.0”, respectively, in the difference between the groups (pH-2.4 g minus Time-2.25 g). The secondary endpoints were the proportion of remission and the proportion of efficacy. Remission was defined as patients with a UC-DAI of 2 or less and a bloody stool score of 0 at the final assessment. Efficacy was defined as remission or improvement. Improvement was defined as patients with the decrease in UC-DAI by two points or more, except patients who experienced a remission. In the safety endpoints the numbers of patients with AEs and patients with ADRs were analyzed by Fisher's exact test.

Unless otherwise specified, differences at α = 0.05 (two-sided) and *P* < 0.05 were considered statistically significant. Differences were considered statistically significant when the 95% CI did not include zero in the difference between two groups. Multiplicity of these analyses was not taken into consideration. The statistical analyses were conducted by ZERIA Pharmaceutical, Japan, based on statistical advice of an expert independent of this study.

The number of patients required to demonstrate the superiority of pH-3.6 g over Time-2.25 g was estimated to be 55 at α = 0.05 (two-sided) and β = 0.1 when the difference between the decreases in UC-DAI of the two groups was 2.0 and the standard deviation (SD) was 3.2. The number of the patients required to demonstrate the noninferiority of pH-2.4 g to Time-2.25 g was estimated to be 54 at α = 0.05 (two-sided), β = 0.1 and Δ = 1 when the difference between the decreases in UC-DAI of the two groups was 1.0 and the SD was 3.2. According to the above estimations, we decided to enroll at least 60 patients in each active-drug group considering the patients excluded from the analysis set. Placebo was used as the reference in the analysis for efficacy, and the number of patients in the placebo group was half of that in each of the active drug groups.

The full analysis set (FAS) consisted of all participants except those who had not taken even one tablet of the investigational drugs, those who did not comply with Good Clinical Practice (GCP), those who met exclusion criteria 1) and those whose data were missing. The per protocol set (PPS) consisted of the FAS except those who did not fulfill the inclusion criteria, those who met the exclusion criteria 2)–7), those who received forbidden drugs and those whose drug compliance was less than 75%. Concerning the withdrawal cases, their adoption was to be decided before the blind was removed. The statistical analysis of efficacy was performed primarily based on data from the FAS followed by comparison with those from the PPS. The dataset for safety consisted of all participants except those who had not taken even one tablet of the investigational drug and those who did not comply with the GCP.

### Independent Image Assessment Committee

We established an image assessment committee independent from the investigators to ensure the reliability of the mucosal appearance scores, and each of the three members of the committee blindly and independently scored mucosal appearance by examining photos provided by the investigators. When the scores obtained from all three members was the same, that score was regarded as a judgment by the committee. If the scores were different, the committe members discussed the case until they reached a consensus. When the judgment by the committee and the evaluation by the investigators were the same, it was defined as an agreement case.

## RESULTS

### Patient Demographics

**FIGURE 1:**
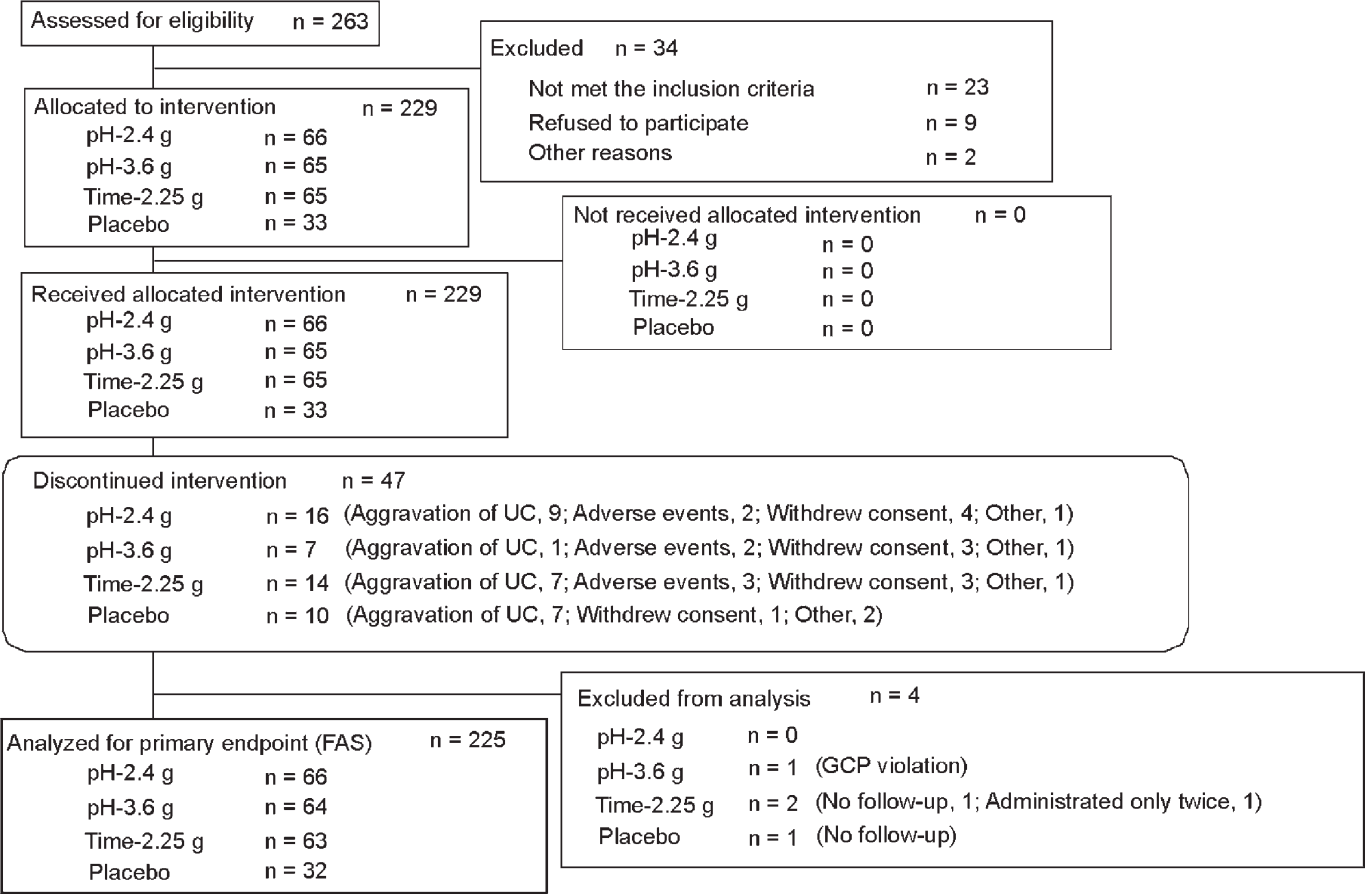
Enrolment, randomization, and follow-up of the study patients.

Investigators obtained informed consent from 263 patients during the period from November 2005 to July 2007 and completed the final follow-up in September 2007 (Fig. 1). Of the 263 patients, 229 patients were assigned to all the groups (pH-2.4 g, 66; pH-3.6 g, 65; Time-2.25 g, 65; Placebo, 33). All 229 patients took the investigational drugs at least once. Drug compliance was greater than 75% in every patient except for 2 patients (Time-2.25 g, 1; Placebo, 1).

A total of 47 patients (pH-2.4 g, 16; pH-3.6 g, 7; Time-2.25 g, 14; Placebo, 10) were withdrawn from the study. The most frequent reason for withdrawal was aggravation of UC (pH-2.4 g, 9; pH-3.6 g, 1; Time-2.25 g, 7; Placebo, 7).

There were 225 patients in the FAS (pH-2.4 g, 66; pH-3.6 g, 64; Time-2.25 g, 63; Placebo, 32) and 222 patients in the PPS (pH-2.4 g, 65; pH-3.6 g, 62; Time-2.25 g, 63; Placebo, 32). The results were very similar when the data were analyzed according to the FAS or PPS. Therefore, the results analyzed according to the FAS will be shown at the following. We did not perform adjustments for the demographic factor because patient demographics in all groups were similar (Table 1).

**TABLE 1:** Patient Demographics

	pH-2.4 g (*n* = 66)	pH-3.6 g (*n* = 64)	Time-2.25 g (*n* = 63)	Placebo (*n* = 32)
Sex (male/female)	38/28	36/28	37/26	16/16
Age (years)				
Mean	39.4	41.6	41.2	35.8
SD	12.0	10.4	10.1	10.6
Weight (kg)				
Mean	59.45	60.20	61.11	59.49
SD.	11.38	9.39	10.92	10.47
Years of disease duration (no. of patients)				
<1	21	16	9	7
<2	7	9	6	5
<3	4	4	5	2
<4	3	2	2	0
<5	4	5	3	3
≥5	27	28	38	15
Inflamed areas (no. of patients)				
Proctitis-type	24	24	25	11
Others	42	40	38	21
Clinical course (no. of patients)				
Initial	16	14	8	5
Relapsed	50	50	55	27
UC-DAI at initial assessment				
Mean	6.1	6.0	6.1	5.9
SD	1.6	1.6	1.6	1.7

### Efficacy

**FIGURE 2:**
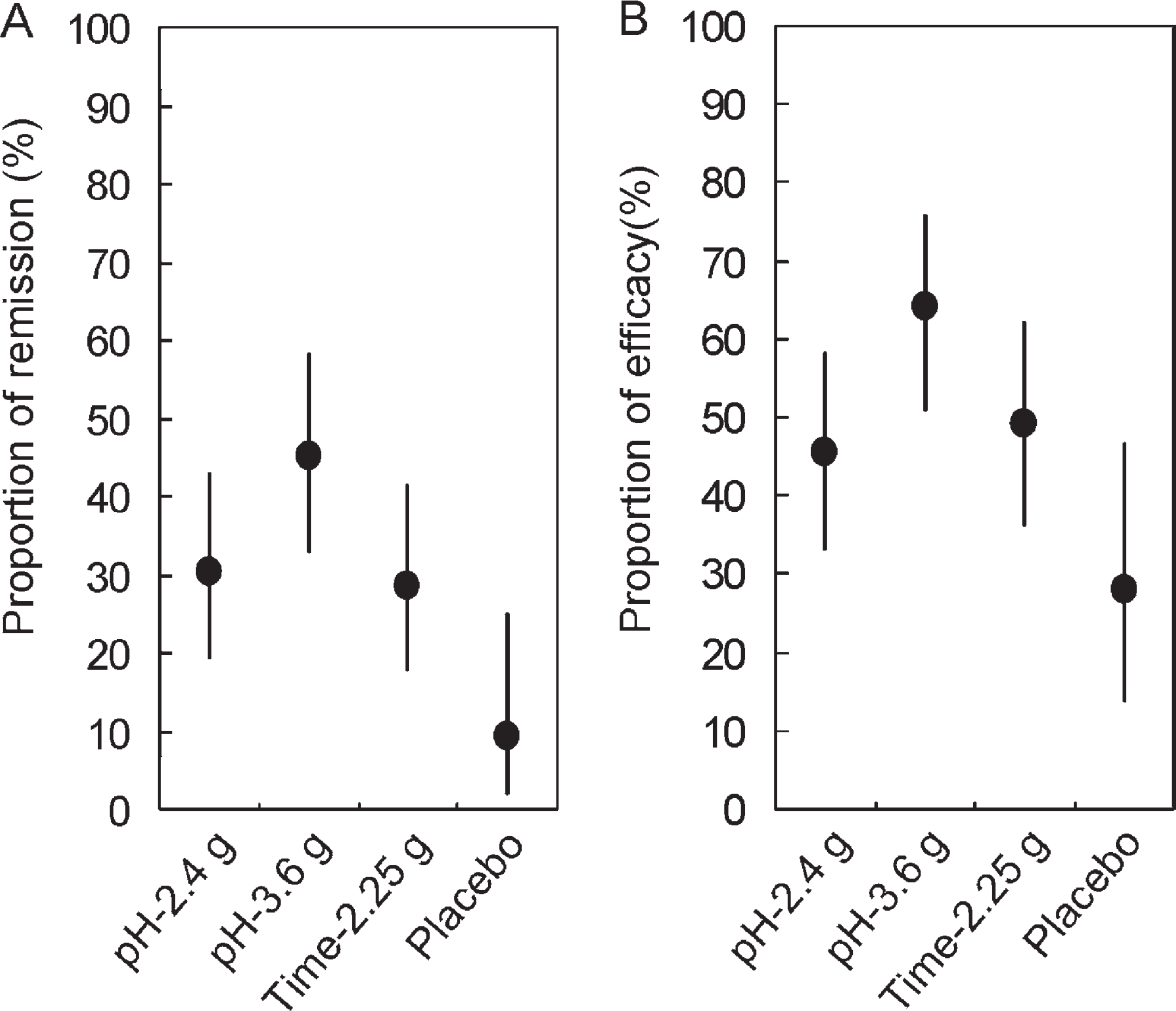
Proportion of remission and efficacy. The graphs show proportions of remission and efficacy in each group within 95% CIs. Each graph includes 225 patients for analyses (pH-2.4 g, 66; pH-3.6 g, 64; Time-2.25 g, 63; Placebo, 32). A: The proportion of patients who experienced a remission was 30.3% (CI, 19.6–42.8) in pH-2.4 g, 45.3% (CI, 32.9–58.2) in pH-3.6 g, 28.6% (CI, 17.9–41.3) in Time-2.25 g, and 9.4% (CI, 2.0–25.0) in Placebo. There were statistically significant differences from Placebo in all active-drug groups. B: Efficacy was archived in 45.5% (CI, 33.2–58.1) in pH-2.4 g, 64.1% (CI, 51.1–75.6) in pH-3.6 g, 49.2% (CI, 36.4–62.1) in Time-2.25 g, and 28.1% (CI, 13.8–46.7) in Placebo. There were significant differences from Placebo in both pH-3.6 g and Time-2.25 g.

The decrease in UC-DAI as the primary endpoint was most pronounced in pH-3.6 g (Table 2). The decrease in UC-DAI was greater by 1.6 (95% CI: 0.6, 2.6) in pH-3.6 g compared to Time-2.25 g, demonstrating the superiority of pH-3.6 g over Time-2.25 g (*P* = 0.003). The difference between pH-2.4 g and Time-2.25 g was 0.2 (95% CI: −0.8, 1.2), demonstrating the noninferiority of pH-2.4 g to Time-2.25 g. The secondary endpoints, specifically the proportions of the remission and efficacy, were the highest in pH-3.6 g (Fig. 2).

**TABLE 2:** Decrease in the UC-DAI

	pH-2.4 g (*n* = 66)	pH-3.6 g (*n* = 64)	Time-2.25 g (*n* = 63)	Placebo (*n* = 32)
Decrease in the UC-DAI				
No. of patients	58	62	60	32
Mean (95% CI)	1.5 (0.7, 2.3)	2.9 (2.3, 3.5)	1.3 (0.6, 2.1)	0.3 (−0.7, 1.2)
Difference from Time-2.25 g (95% CI)	0.2 (−0.8, 1.2)	1.6 (0.6, 2.6)	—	—
Difference from Placebo (95% CI)	1.2 (0.0, 2.5)	2.7 (1.4, 3.9)	1.1 (−0.1, 2.3)	—

Decrease in the UC-DAI was calculated from the scores at the initial and final assessments. The data of 13 patients (pH-2.4 g, 8; pH-3.6 g, 2; Time-2.25 g, 3) had to be excluded from the analysis because the mucosal appearance data were missing.

In the subgroup analyses, a significant difference in the decrease in UC-DAI was found between pH-3.6 g and placebo in all subgroups (Table 3). Among the patients with proctitis-type UC, there were significant differences both in pH-2.4 g and in pH-3.6 g as compared with placebo, but not in Time-2.25 g as compared with placebo. Among the patients in which UC-DAI at initial assessment was “3–5”, the results showed a trend similar to the subgroup of patients with proctitis-type UC. On the other hand, among the cases where UC-DAI was “6–8”, there was a significant difference only in pH-3.6 g as compared with placebo, but not in pH-2.4 g and Time-2.25 g as compared with placebo.

**TABLE 3:** Subgroup Analysis: Inflamed Areas and Severity

		pH-2.4 g (*n* = 66)	pH-3.6 g (*n* = 64)	Time-2.25 g (*n* = 63)	Placebo (*n* = 32)
Decrease in UC-DAI					
Inflamed areas					
Proctitis-type	No. of patients	22	23	23	11
	Mean (95% CI)	1.8 (0.7, 2.8)	1.7 (0.7, 2.6)	1.1 (-0.2, 2.3)	−0.4 (-1.8, 1.1)
	Difference from Time-2.25 g (95% CI)	0.7 (-0.8, 2.1)	0.6 (-0.9, 2.0)	—	—
	Difference from Placebo (95% CI)	2.1 (0.3, 4.0)	2.0 (0.2, 3.8)	1.5 (-0.4, 3.3)	—
Others	No. of patients	36	39	37	21
	Mean (95% CI)	1.3 (0.2, 2.4)	3.6 (2.9, 4.4)	1.5 (0.5, 2.5)	0.6 (-0.8, 1.9)
	Difference from Time-2.25 g (95% CI)	−0.2 (-1.5, 1.2)	2.2 (0.8, 3.5)	—	—
	Difference from Placebo (95% CI)	0.7 (-0.9, 2.4)	3.1 (1.5, 4.7)	0.9 (-0.7, 2.5)	—
UC-DAI at initial assessment					
3-5	No. of patients	23	27	24	13
	Mean (95% CI)	1.7 (0.8, 2.6)	1.8 (0.9, 2.7)	1.5 (0.3, 2.6)	−0.1 (-1.5, 1.3)
	Difference from Time-2.25 g (95% CI)	0.2 (-1.1, 1.6)	0.4 (-1.0, 1.7)	—	—
	Difference from Placebo (95% CI)	1.8 (0.1, 3.4)	1.9 (0.3, 3.5)	1.5 (-0.1, 3.2)	—
6-8	No. of patients	35	35	36	19
	Mean (95% CI)	1.3 (0.1, 2.5)	3.7 (2.9, 4.6)	1.2 (0.2, 2.3)	0.5 (-0.9, 1.9)
	Difference from Time-2.25 g (95% CI)	0.1 (-1.3, 1.6)	2.5 (1.1, 4.0)	—	—
	Difference from Placebo (95% CI)	0.9 (-0.9, 2.6)	3.3 (1.5, 5.0)	0.7 (-1.0, 2.5)	—

Decrease in the UC-DAI was calculated from the scores at the initial and final assessments. The data of 13 patients (pH-2.4 g, 8; pH-3.6 g, 2; Time-2.25 g, 3) had to be excluded from the analysis because the mucosal appearance data were missing.

### Reliability of the Mucosal Appearance Scores

The proportion of agreement between the judges by the image assessment committee and the evaluations by the investigators are summarized in Table 4. The proportion was 67.9%, and Cohen's κ coefficient was 0.497. In all the disagreement cases there was one degree of difference in the scores between the judges by the committee and the evaluations by the investigators.

**TABLE 4:** Agreement Between Evaluations by the Investigators and Judgments by the Image Assessment Committee

		Evaluations by the Investigators					
*n* = 193		0	1	2	3	Total	
Judgments by committee	0	26	9	0	0	35	Proportion of agreement (%) 67.9
	1	11	63	23	0	97	
	2	0	14	41	5	60	
	3	0	0	0	1	1	
	Total	37	86	64	6	193	Cohen's κ coefficient 0.497

Proportion of agreement (%) = (number of agreement cases) / (number of cases confirmed by colonoscopy) × 100

In this trial, 229 patients were allocated to an intervention. The data of 35 patients had to be excluded from the analysis because the mucosal appearance score was missing, and the data of one patient had to be excluded from the analysis because of a GCP violation.

### Safety

In patients with AEs and ADRs, there were no significant differences between each of the active drug groups and placebo (Table 5). Serious adverse events included aggravation of UC in 2 patients in pH-2.4 g, malaise in 1 patient in pH-3.6 g, abdominal abscess in 1 patient in pH-3.6 g and aggravation of UC in 3 patients in Time-2.25 g. A causal relationship to the drug could not be ruled out in 3 patients with serious AEs (1 patient in pH-2.4 g and 2 patients in Time-2.25 g).

**TABLE 5:** Adverse Events and Adverse Drug Reactions

	pH-2.4 g (*n* = 66)	pH-3.6 g (*n* = 64)	Time-2.25 g (*n* = 65)	Placebo (*n* = 33)
	
	No. of Patients (%)	No. of Patients (%)	No. of Patients (%)	No. of Patients (%)
Adverse events*	56 (84.8)	53 (82.8)	55 (84.6)	22 (66.7)
Nasopharyngitis	11 (16.7)	10 (15.6)	7 (10.8)	2 (6.1)
C-reactive protein increased	13 (19.7)	14 (21.9)	18 (27.7)	6 (18.2)
Beta-N-acetyl-D-glucosaminidase increased	13 (19.7)	12 (18.8)	13 (20.0)	6 (18.2)
Eosinophil count increased	12 (18.2)	9 (14.1)	14 (21.5)	4 (12.1)
Lymphocyte count decreased	10 (15.2)	5 (7.8)	11 (16.9)	1 (3.0)
Blood bilirubin increased	6 (9.1)	8 (12.5)	5 (7.7)	4 (12.1)
White blood cell count increased	4 (6.1)	4 (6.3)	7 (10.8)	2 (6.1)
Blood lactate dehydrogenase increased	2 (3.0)	0 (0.0)	6 (9.2)	4 (12.1)
Adverse drug reactions	27 (40.9)	31 (48.4)	28 (43.1)	10 (30.3)

* Events that occurred in more than 10% of the patients in at least one group.

## DISCUSSION

Previous randomized controlled studies have stated that the pH- and time-dependent release formulations of mesalamine used in the present study were superior to placebo.^9^,^11^ However, exposure periods and dosage differed in the individual studies. These differences made it difficult to compare drug efficacies.

In the present study the pH-3.6 g group showed the highest efficacy at every endpoint. In general, it has been proposed that mesalamine exerts a greater therapeutic effect at higher doses.^1^,^2^,^12^ Schroeder et al^13^ demonstrated that a higher dose of the pH-dependent release formulation of 4.8 g/day showed greater efficacy than a dose of 1.6 g/day. In addition, when another formulation of pH-dependent release mesalamine (Rowasa) was administered to patients with active UC, UC-DAI after six weeks of treatment decreased from 8.5 to 4.8 at the 4 g/day dose, from 9.0 to 7.7 at the 2 g/day dose, and from 8.2 to 7.7 in the placebo group.^14^ In this report, we also showed that the pH-dependent release formulation at a higher dose significantly decreased UC-DAI (Table 2).

Patients with proctitis-type UC account for 40–50% of the patients with UC.^12^ Physicians often use mesalamine suppositories and enemas to treat these patients, but compliance is poorer than with oral formulations. We found significant differences both in the pH-2.4 g and in the pH-3.6 g group as compared with placebo in patients with “proctitis-type” disease, but not in the Time-2.25 g group (Table 3). These findings suggest that the pH-dependent release formulation is preferable for patients with inflammation of the distal intestine. Until now, mesalamine formulations have been chosen without adequate supporting evidence. The results of the present study provide scientific evidence for the proper use of the different mesalamine formulations, especially when the location of the inflammation is taken into consideration.

In every subgroup of disease characteristics, only the pH-3.6 g group showed significant differences as compared with placebo (Table 3). Especially in subgroups classified as “others” and as UC-DAI of “6–8,” the pH-3.6 g group showed greater a decrease in UC-DAI compared to the other groups. We presume that patients who are classified as UC-DAI of “6–8” (i.e., the patients with more severe disease) can only achieve an adequate level of mesalamine at the dosage of 3.6 g/day. As a result, a substantial change in the UC-DAI score may occur. Patients with more extensive disease generally have more severe disease.^12^ Therefore, because the patients classified as “others” had more severe disease than the patients with the “proctitis-type” UC, a substantial change in UC-DAI might be also observed in patients classified as “others.”

In the primary endpoint, neither the pH-2.4 g group nor Time-2.25 g group showed significant differences when compared with placebo (Table 2). These results were in contrast to the findings from previous studies.^9^,^11^ This discrepancy was likely related to the lack of power; i.e., the number of patients in placebo was half of that in the active drug groups because the present study was not designed mainly for comparing the active drug groups with placebo.

The proportion of agreement between the judges by committee and the evaluations by the investigators was 67.9% (Cohen's κ coefficient: 0.497) (Table 4). Mucosal appearance in patients with UC is often evaluated using the Baron score,^15^ which is similar to the index used in our study. Hirai and Matsui^16^ reported a proportion of agreement between the scores of the two raters that used the Baron score. In their study, the proportion of agreement and κ coefficient between two raters were 51% and 0.31, respectively, but their coefficient was lower compared to our study. In the Hirai and Matsui study, 8.7% of all patients observed two degrees of difference in the scores between two raters. On the other hand, in our study, there were no cases showing two degrees of difference. Thus, we assumed that the interobserver variation among the investigators was well controlled in our study.

In the present study, the proportion of cases showing disagreement was ≈30%. This disagreement was predominately ascribed to a difference in the approach to the patients' mucosa, because the investigators evaluated the actual mucous membrane through colonoscopy; whereas the committee used photos of the mucous membrane taken by the investigators. We provided atlases of mucosal appearance to the investigators in order to minimize interobserver variation (both investigator–investigator and investigator–committee). However, as mentioned by Hirai and Matsui,^16^ further improvement in the agreement between raters could be accomplished by better training of the raters, the use of good photos, and so on.

Mesalamine has generally been found to be a safe drug,^1^,^2^ but the transfer of mesalamine from the upper gastrointestinal tract to the plasma should be minimized considering its nephrotoxicity. We expect the pH-dependent release formulation to reduce the frequency of AEs because it suppresses the transfer of mesalamine to plasma due to its release mechanism.^3^,^4^ However, no difference between the two formulations in the proportion of the patients with AEs was observed (Table 5). The wide safety margin of mesalamine may blur the differences between the two formulations.

In summary, this is the first study to directly compare the efficacy and safety of two different mesalamine formulations for the induction of remission in patients with UC. The results of our study clearly showed superior efficacy of the pH-dependent release formulation administered at a dose of 3.6 g/day and superior characteristics of this formulation to treat patients with proctitis-type UC. However, it is unknown whether the formulation is also efficacious in patients with more severe UC because the subjects in this study were patients with mild-to-moderate active UC. Mesalamine is considered safer than corticosteroids. Accordingly, further research will be necessary to fully evaluate the role of mesalamine formulations for the treatment of severe UC. If this is accomplished, it is likely that mesalamine contributes to the improvement in the quality of life of patients with UC.
